# Ocular Thelaziosis in Dogs, France

**DOI:** 10.3201/eid1612.100872

**Published:** 2010-12

**Authors:** Perrine Ruytoor, Eric Déan, Olivier Pennant, Philippe Dorchies, René Chermette, Domenico Otranto, Jacques Guillot

**Affiliations:** Author affiliations: École Nationale Vétérinaire d’Alfort, ANSES, UPEC, Maisons-Alfort, France (P. Ruytoor, R. Chermette, J. Guillot);; Clinique Vétérinaire, Lognes, France (E. Déan);; Clinique Vétérinaire, Vergt, France (O. Pennant);; É́cole Nationale Vétérinaire de Toulouse, Toulouse, France (P. Dorchies);; University of Bari, Bari, Italy (D. Otranto)

**Keywords:** Thelazia callipaeda, ocular thelaziosis, France, parasites, parasitic diseases, Dordogne, dog, eyes, dispatch

## Abstract

During 2005–2008, veterinary practitioners reported ocular infection by *Thelazia* spp. nematodes in 115 dogs and 2 cats in southwestern France. Most cases were detected in Dordogne, particularly in 3 counties with numerous strawberry farms, which may favor development of the fruit fly vector. Animal thelaziosis may lead to emergence of human cases.

*Thelazia* spp. (Spirurida, Thelaziidae) nematodes live in the conjunctival sac of warm-blooded vertebrates. These nematodes are responsible for epiphora, conjunctivitis, keratitis, and corneal ulcers (*1**–**3*). *Thelazia* spp. nematodes are transmitted by different species of flies feeding from the lacrimal secretions of the definitive hosts. Among the 10 species, *T. californiensis* and *T. callipaeda* parasitize carnivores and sometimes humans. *T. californiensis* is confined to the western United States and has never been reported in Europe (*1*). *T. callipaeda*, the “oriental eye worm,” is common in the former Soviet republics and in India, Thailand, People’s Republic of China, and Japan (*2*), where it causes infections in humans, dogs, and cats (*3*). Wild mammals, such as foxes and lagomorphs, are reservoir hosts for the nematodes. During the past decade *T. callipaeda* infection was proven to be widespread among dogs and cats from northern (Aosta valley) to southern (Basilicata region) Italy (*4*). In Ticino, a region of southern Switzerland, a retrospective study identified 106 *T. callipaeda*–positive dogs and 5 positive cats during 2005–2007 (*5*). Recently, the first autochthonous case of thelaziosis in a dog was described in southern Germany (*6*). Locally transmitted cases of thelaziosis were first reported in 4 dogs and 1 cat that lived or spent time in the department of Dordogne in southwestern France (*7*).

## The Study

**Figure 1:**
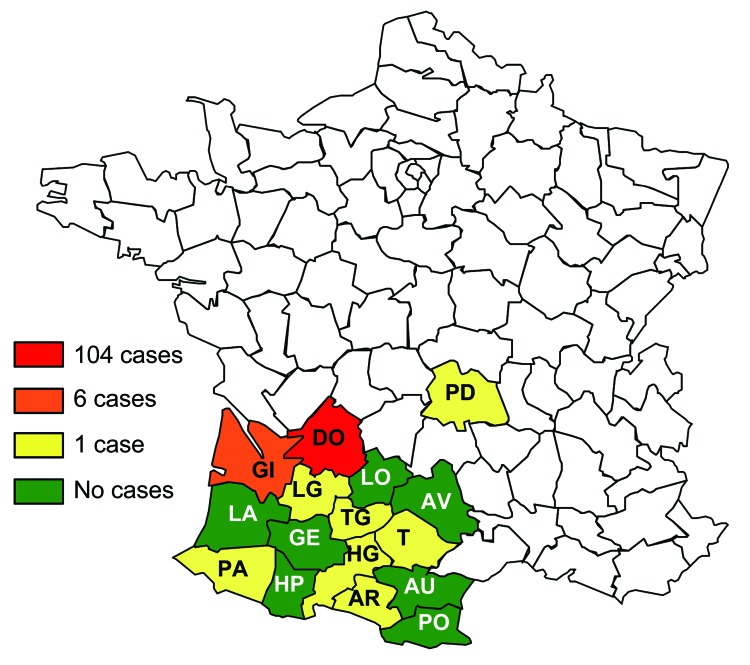
Departments in which the epidemiologic survey for thelaziosis was conducted and number of cases of canine and feline thelaziosis, France, 2005–2008. Clinical cases of thelaziosis were reported in 9 departments. PO, Pyrénées-orientales; AR, Ariège; AU, Aude; AV, Aveyron; DO, Dordogne; GE, Gers; GI, Gironde; HG, Haute-Garonne; HP, Hautes-Pyrénées; LA, Landes; LG, Lot-et-Garonne; LO, Lot; PA, Pyrénées-Atlantiques; PD, Puy-de-Dôme; T, Tarn; TG, Tarn-et-Garonne.

At the end of 2008, we contacted veterinary practitioners from 938 veterinary practices in 16 departments in France by regular mail. The survey covered a large part of southwestern France (Figure 1), where the first thelaziosis cases in dogs and cats were reported in 2007 (*7*). Veterinary practitioners were asked whether they had diagnosed ocular thelaziosis in a dog or a cat during the previous 3 years. For each clinical case, a short questionnaire asked for a description of the animal (i.e., sex, age, breed, use), description of the place where the animal lived, and treatment protocol.

**Figure 2:**
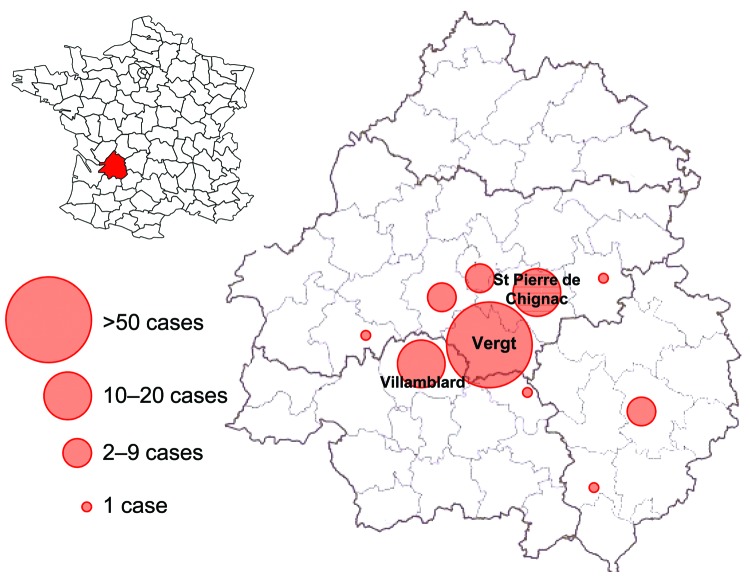
Department of Dordogne (with its 4 arrondissements and 50 counties) and distribution of clinical cases of thelaziosis in dogs and cats, France, 2005–2008. Most cases were reported in the counties of Vergt, Saint Pierre de Chignac, and Villamblard.

A total of 117 clinical cases of thelaziosis (115 dogs and 2 cats) was reported in 22 veterinary practices from 9 departments (Ariège, Dordogne, Gironde, Haute-Garonne, Lot-et-Garonne, Puy-de-Dôme, Pyrénées-Atlantiques, Tarn, and Tarn-et-Garonne). Most (104 [89%]) cases were diagnosed from 10 practices in Dordogne (Figure 1). In each of the other departments, only a few (1–6) cases were diagnosed. Furthermore, most of the infected animals in other departments had spent time in Dordogne a few months before clinical signs developed. In Dordogne, most cases were from the center of the department, with 3 counties overrepresented (60 cases in Vergt, 16 cases in Saint-Pierre-de-Chignac, and 9 cases in Villamblard) (Figure 2). In these counties, strawberry production is predominant and may favor development of the fruit fly vector, *Phortica variegata*; in other areas of Dordogne, other types of fruit production (plum or apple) are reported. All infected dogs and the cats were 6 months–14 years of age and privately owned. Ninety-one (78%) of the 117 animals lived in a small village; 22 were farm dogs. Twenty-six animals lived in a city, but all had free access to the outdoors.

The animals were referred to veterinary practitioners for unilateral or bilateral conjunctivitis. For all animals, nematodes were observed on the eye surface. The first cases of thelaziosis were detected in 2 dogs and 1 cat in the county of Vergt at the end of 2005. During 2006, a total of 27 cases were detected in late summer and autumn; animals may have been contaminated by infected vectors during the peak of the male *Phortica* spp. fly population in summer 2006. During 2007 and 2008, clinical cases were detected throughout the year. The apparent absence of seasonality for detecting adult *Thelazia* spp. nematodes in definitive hosts is in accordance with previous observations in areas in Italy to which *Thelazia* spp. nematodes are endemic (*8*).

Nematodes were collected from the eyes of 19 dogs and 1 cat and morphologically identified according to Skrjabin et al. (*9*). To determine the haplotype sequence, we processed specimens using the specific amplification of a partial sequence of the mitochondrial cytochrome c oxidase subunit 1 gene (*cox1*, 605 bp), as previously described (*10*). The sequences obtained were identical to the sequence representing haplotype 1 of *T. callipaeda* (GenBank accession no. AM042549) previously reported in Italy and Switzerland but they displayed a 1.3%-nt difference from the haplotype recently detected in Germany (*6*).

## Discussion

Before 2005, thelaziosis had been reported only sporadically in France (*11**,**12*), occurring in dogs that had spent time during summer in northern Italy. In 2007, Dorchies et al. described 5 locally transmitted thelaziosis cases from southwestern France (*7*). The present investigation indicates that Dordogne and, more precisely, the county of Vergt should now be considered as an area to which ocular thelaziosis is endemic. This area is near the Atlantic Ocean and is part of the Aquitaine Region (44°–45°N, ≈0°). Its altitude ranges from 112 m to 246 m, and it has an oceanic climate with an average of 800 mm annual rainfall. This area is at the same latitude as Aosta valley in northern Italy, where thelaziosis in dogs is regularly reported. It belongs to the putative areas in which the drosophilid species *P. variegata*, the *T. callipaeda* vector, could be present according to a predictive geoclimatic model in Europe (*13*).

The *T. callipaeda* nematode may have been introduced in France by importation or dispersal of vectors and/or reservoir hosts. The dispersal of infected vectors is unlikely because fruit flies are not as robust as other vectors, such as mosquitoes, and are not known to disperse by wind. Introduction by an infected animal seems to be more likely. Adult parasites may have been introduced by a dog (or a small number of dogs) that spent time in a thelaziosis-endemic area in Italy or southern Switzerland during 2005. Another explanation for the introduction of thelaziosis in Dordogne would be migration of infected wild animals (such as foxes) from Switzerland or Italy. However, Dordogne is far from these areas (500 km–600 km) and separated by the Alps. In such circumstances, the possibility of population exchanges is limited. Our final explanation could be the importation of wild hares for hunting in Dordogne. Introduction of infected hares from Italy already has been implicated in outbreaks of animal and human cases of tularemia in Dordogne (*14*). A recent investigation in southern Italy demonstrated the existence of an active sylvatic life cycle of *T. callipaeda* nematodes (*15*). Further studies in the county of Vergt should include investigation of eye worms in wild mammals.

## Conclusions

Once introduced in Dordogne, this parasite might have found appropriate conditions for the perpetuation of its life cycle. Our investigation showed that cases in companion animals were located where strawberry production was predominant. *T. callipaeda* nematodes may be transmitted to humans, and animal thelaziosis in Dordogne may lead to emergence of human cases.
